# Inhibition of kinase IKK*β* suppresses cellular abnormalities induced by the human papillomavirus oncoprotein HPV 18E6

**DOI:** 10.1038/s41598-020-80193-5

**Published:** 2021-01-13

**Authors:** Mojgan Padash Barmchi, Miranda Thomas, Jayashree V. Thatte, Arushi Vats, Bing Zhang, Ross L. Cagan, Lawrence Banks

**Affiliations:** 1 Department of Biology, University of Oklahoma, Norman, OK USA; 2 International Centre for Genetic Engineering and Biotechnology, Trieste, Italy; 3 Biotech Research and Innovation Centre, University of Copenhagen, Copenhagen, Denmark; 4 Division of Biological Sciences, University of Missouri, Columbia, MO USA; 5 Institute of Cancer Sciences, University of Glasgow, Wolfson Wohl Cancer Research Centre, Glasgow, Scotland UK

**Keywords:** Cancer, Cell biology, Microbiology

## Abstract

Human papillomavirus (HPV) is the leading cause of cervical cancer and has been implicated in several other cancer types including vaginal, vulvar, penile, and oropharyngeal cancers. Despite the recent availability of a vaccine, there are still over 310,000 deaths each year worldwide. Current treatments for HPV-mediated cancers show limited efficacy, and would benefit from improved understanding of disease mechanisms. Recently, we developed a *Drosophila* ‘HPV 18 E6’ model that displayed loss of cellular morphology and polarity, junctional disorganization, and degradation of the major E6 target Magi; we further provided evidence that mechanisms underlying HPV E6-induced cellular abnormalities are conserved between humans and flies. Here, we report a functional genetic screen of the *Drosophila* kinome that identified IKK$\beta$—a regulator of NF-κB—as an enhancer of E6-induced cellular defects. We demonstrate that inhibition of IKK$\beta$ reduces Magi degradation and that this effect correlates with hyperphosphorylation of E6. Further, the reduction in IKK$\beta$ suppressed the cellular transformation caused by the cooperative action of HPVE6 and the oncogenic Ras. Finally, we demonstrate that the interaction between IKK$\beta$ and E6 is conserved in human cells: inhibition of IKK$\beta$ blocked the growth of cervical cancer cells, suggesting that IKK$\beta$ may serve as a novel therapeutic target for HPV-mediated cancers.

## Introduction

Human papillomaviruses (HPV) are small, double-stranded circular DNA viruses that are obligate epitheliotrophs: they specifically infect either squamous or mucocutaneous epithelia. The latter HPV types can be categorized into two classes: low-risk HPVs and high-risk HPVs. The former induce large and obvious benign lesions—condylomata—of the infected tissues. The latter are carcinogenic, causing most cervical cancers, as well as being strongly implicated in oropharyngeal, anal, vulvar, vaginal and penile cancers^1^. Among all high-risk HPVs, HPV 16 and HPV 18 are the most prominent types, causing more than 70% of all invasive cervical cancers. According to World Health Organization (WHO), every year 570,000 women are diagnosed with cervical cancer, with a current mortality rate of more than 50%^2,3^.

The availability of effective vaccines against the most prevalent high-risk HPVs is expected to eventually reduce HPV-dependent tumors. However, the number of new HPV-induced cancer cases is not predicted to decline appreciably for the next few decades. Economic and cultural barriers hinder widespread immunization in the middle- and low-income countries that account for the majority of cervical cancers^4^. Furthermore, chronic HPV infection can require several decades to provoke transformation^5,6^. Existing vaccines are only prophylactic against new HPV infections and are not effective against preexisting HPV infections, nor can they inhibit cancer progression and malignancy^7^. As a result of these hurdles, effective treatment for HPV-induced cancer in general remains a major unmet clinical need. Current treatments for invasive HPV-induced cancers are primarily radiation and chemotherapy, which show limited effectiveness and the survival rates of, for example*,* advanced-stage cervical cancer patients is low^8,9^.

HPV 16 and HPV 18 direct cellular transformation through the persistent expression of two viral early genes, E6 and E7^10,11^. E6 and E7 oncoproteins cause cellular transformation through elimination of key tumor suppressors P53, and Rb respectively^12–14^. High-risk HPV E6s contain a PDZ binding motif (PBM) at the extreme C terminus that is absent in low-risk E6s^15^. Interaction of the PBM with the PDZ domains of key host cellular PDZ domain proteins, including Magi, Dlg and Scribble, targets these proteins for ubiquitination and subsequent proteasome-mediated destruction^16–19^. These PDZ domain-containing proteins are important components of tight junctions and cell polarity-controlling complexes^20–22^. This action of E6 requires the assistance of the host E3 ubiquitin ligase, UBE3A and is necessary for cellular transformation^23,24^. Transgenic mice expressing high-risk E6 and E7 in the skin develop cancers at high frequency; those expressing E7 and an E6 deficient in the PBM do not develop cancers. The failure to induce cellular transformation is independent of P53, as PBM-deleted E6 retains the ability to inactivate P53^25^. However, beyond the cellular targets of HPV oncogenes, we have a limited understanding of how persistent expression of E6 and E7 can lead to dysplasia and cancer.

*Drosophila* has proven a strongly useful platform for modeling human diseases including cancer, owing in part to high conservation of genes and signaling pathways, and the availability of a broad array of genetic tools^26–31^. One particular advantage of *Drosophila* disease models is their use in functional genetic screens designed to discover novel targets and pathways that mediate human disease^32–34^. In this study we used a recently developed *Drosophila* model of HPV18 E6 + UBE3A^35^ in a screen to identify kinases active in aspects of E6/UBE3A-induced transformation. We report that reduced activity of ‘inhibitor of nuclear factor kappa-B kinase subunit beta’ (IKKβ)—which regulates the innate immune response—strongly suppressed E6 + UBE3A-mediated cellular abnormalities, as well as rescuing degradation of E6 targets. We provide evidence that the IKK$\upbeta$-mediated suppression of Magi degradation is due to phosphorylation of E6, which was previously shown to block the interaction of E6 with PDZ domain proteins. Further, reduction in IKK$\beta$ suppressed the cellular transformation caused by the cooperative action of HPVE6 and oncogenic Ras. Finally, we used a targeted inhibitor to demonstrate that reduced IKKβ activity results in strongly reduced growth of cultured human cervical cancer cells of established cell lines. Together, our results support the view that targeting IKKβ is a candidate strategy for developing novel lead therapeutics for HPV-induced cancer.

## Material and methods

### Fly strains

The following fly stocks were used: UAS-hUBE3A^36^, UAS-HPV18-E6^35^, GMR-Gal4, Kinome set, IKK$\beta$^1^/TM6B,Tb, UAS-IKK$\beta$ RNAi attp40, UAS-Ras64BV14 from Bloomington *Drosophila* stock center.

### In vitro assays

To examine the effect of IMD 0354 on the growth of HaCat (HPV−ve), HeLa (HPV 18 +ve), CaSki and SiHa (both HPV 16 +ve), the cells were plated out and allowed to adhere overnight. Next day their culture medium was replaced and different concentrations of IMD 0354 (Santa Cruz Biotechnologies, Santa Cruz, CA) (final concentrations 250, 500, 750, 1000, 1250 nM, using DMSO to equalize input volumes and as negative control 0 nM) were added to the plates. Cells were counted 48 h later using a haemocytometer. The assay was repeated 3 times. One-way ANOVA test using the Prism program was used for statistical analysis.

### Cell cycle and apoptosis analysis

HaCat, HeLa and CaSki cells were plated out at equal density, and allowed to attach overnight. The next day they were treated with 500 nM IMD354 (or DMSO alone) for 3 h, then harvested by typsinisation and centrifugation (the medium and all washes were also centrifuged to collect any floating or dead cells). The cell pellet was resuspended in citrate buffer with 0.1% NP-40, RNase and Propidium iodide, and incubated for 30 min in the dark at room temperature. FACS analyses were then performed (FACScalibur, Becton Dickinson) and the cell cycle profiles were analysed (10,000 events per run). The assays were performed four times. Analysis: the % of events recorded in the subG1 (Essentially apoptosis), G1, S and G2 peaks for each run were compared between treated and untreated samples for each cell type and the fold change with treatment was calculated.

### Western blot analysis

HPV18-positive HeLa cells were seeded on 6 cm dishes and allowed to attach overnight. Fresh medium was then added, containing either 0, 100 nM or 500 nM IMD 0345 (in DMSO). After 5 h the cells were harvested in 2× SDS-PAGE gel loading buffer, run on SDS-PAGE, and analyzed by Western Blot. The membrane was probed with antibody specific for phosphorylated E6, as described previously^37,38^. Following incubation with primary antibody, the appropriate horseradish peroxidase (HRP)-conjugated secondary antibodies (Dako) were used, followed by enhanced chemiluminescence (ECL) detection according to the manufacturer's instructions (GE Healthcare). Original blots provided in supplementary information.

### Immunohistochemistry

For immunolabeling pupal eyes, 40–42 h after puparium formation, were dissected in PBS and fixed in 4% formaldehyde. Fixed tissues were washed three times in PBS solution containing 0.1% Triton-X-100 and blocked in 5% normal goat serum for 1 h before incubation with primary antibodies. The primary antibodies used in this study were rabbit anti-Magi 1:200^39^, rabbit anti-Baz 1:1000, rat anti-DE-cadherin 1:50 (Developmental Studies Hybridoma Bank). The appropriate secondary antibodies were conjugated Alexa488, Alexa594, and Alexa 647 (Invitrogen).

## Results

### Kinome screen identified IKKβ as a mediator of E6 + hUBE3A-mediated defects

The fly eye is a compound eye consisting of 750 unit eyes. These ‘ommatidia’ are clusters of sensory neurons arranged in a precisely repeated hexagonal pattern formed by the precise arrangement of supporting pigment cells. Formation of this highly organized pattern requires precisely regulated cell proliferation, cell differentiation and programmed cell death; disruption of any of these processes leads to a disorganized, rough eye phenotype that is readily scored under a light microscope^40^.

**Figure 1:**
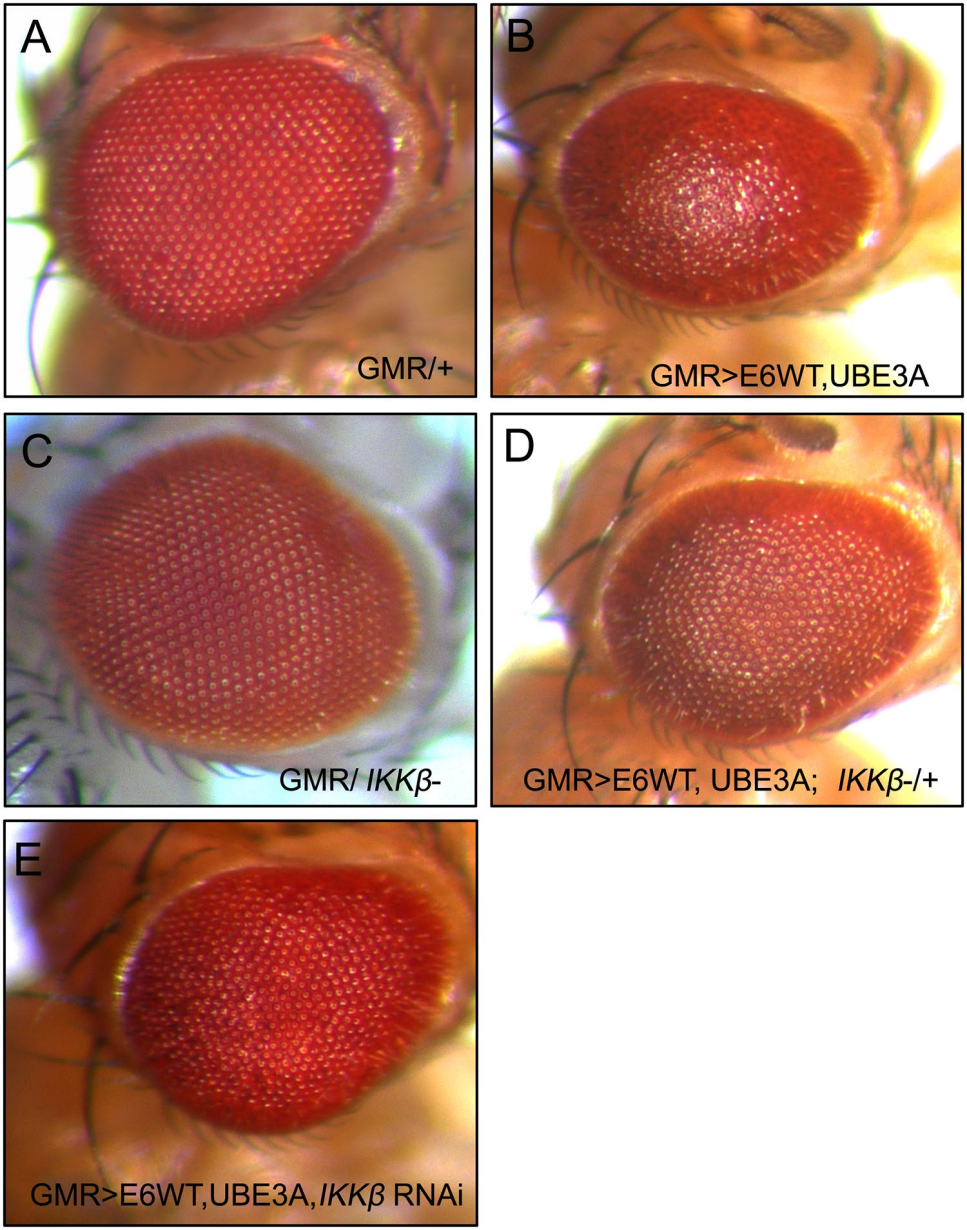
Reduction of IKKβ suppresses the E6 and UBE3A co-expression phenotypes. (**A**) Adult eye expressing GMR-Gal4 showing an intact eye morphology. (**B**) GMR-Gal4-driven co-expression of E6 and UBE3A causes rough eye morphology. (**C**) One mutated copy of IKKβ gene in GMR-Gal4 expressing eye has no effect on the eye morphology. (**D**) One mutated copy of IKKβ gene suppresses the E6 + UBE3A-induced rough eye defects. (**E**) Expression of IKKβ RNAi suppresses the E6 + UBE3A-induced rough eye defects.

We have previously shown that co-expression of E6 and human UBE3A (hUBE3A) in the developing fly eye leads to a disorganized, rough eye phenotype. To identify loci that modify the E6 + hUBE3A-induced eye defects, we used mutations in the kinome to perform a dominant genetic modifier screen. Flies with stable integration of the transgenes *GMR-Gal4*, *UAS-E6*, and *UAS-hUBE3A* (referred to as *GMR*>*E6/hUBE3A*) were crossed to flies heterozygous for a mutant kinase. Comparing *GMR*>*E6/hUBE3A; kinase*^+*/−*^ to *GMR*> *E6/hUBE3A* flies, we screened 195 kinases and identified IKKβ as the strongest suppressor of the E6 + hUBE3A-mediated rough eye phenotype (Fig. 1D, compare with Fig. 1B; Fig. 1A,C are controls). Rescue was evident in 100% of flies (n = 40) with *GMR*>*E6/hUBE3A; IKKβ*^+*/−*^ genotype and to the same extent shown in Fig. 1D. The rescue by reduced IKKβ activity was further confirmed with an RNA-interference transgene targeting IKKβ (Fig. 1E).

### Inhibiting IKKβ in cervical cancer cells blocked their growth

**Figure 2:**
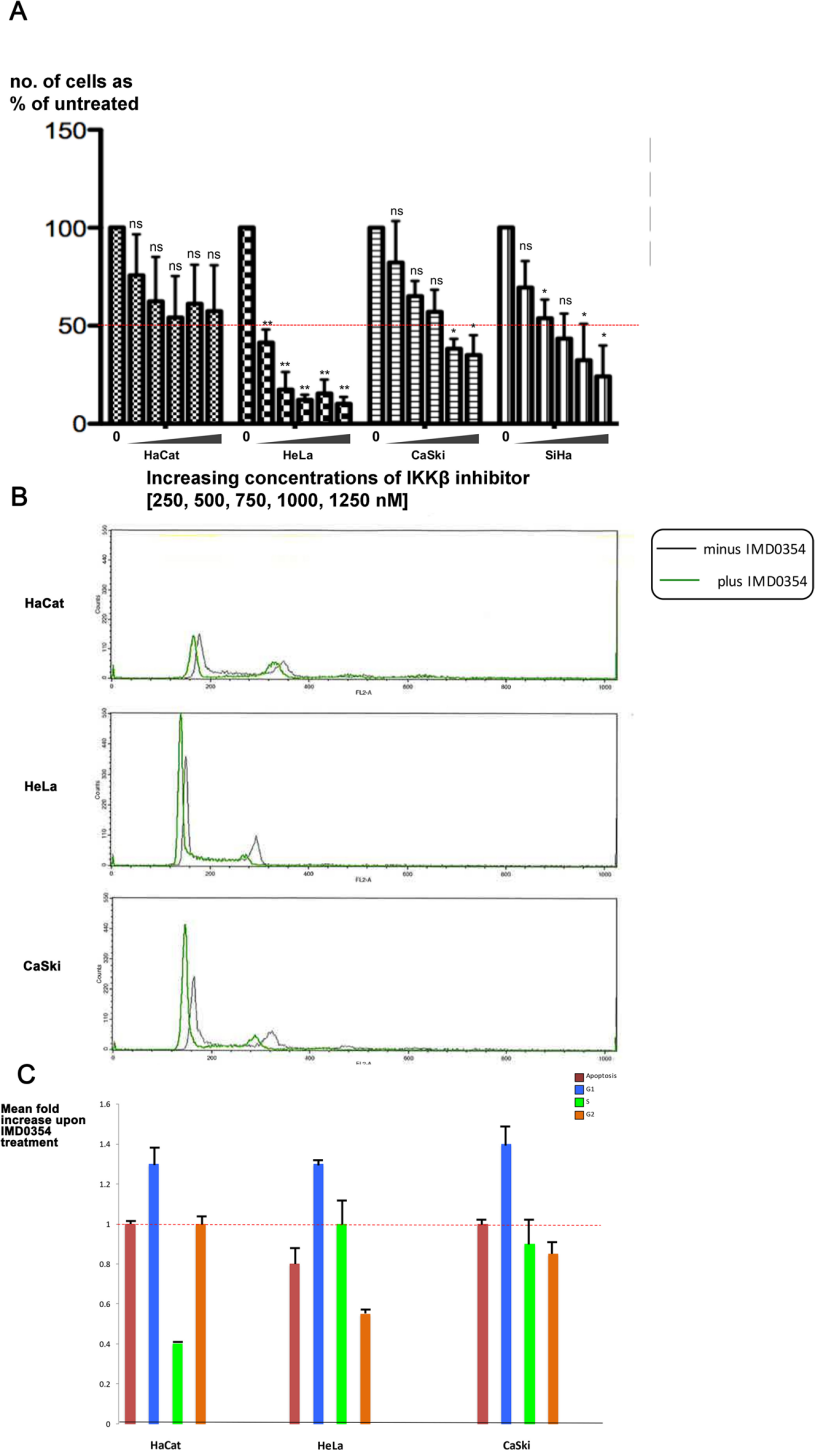
Inhibition of Human IKKβ in cervical cancer cells blocks their growth. (**A**) Five different concentrations (250, 500, 750, 1000, 1250 nM) of human IKKβ inhibitor, IMD 0354, were applied to HPV-negative (HaCat), HPV 18-positive (HeLa), and HPV 16-positive (CaSki and SiHa) cells and the effect was measured by number of surviving cells after 48 h. The inhibitor blocks the growth of HPV 16 and 18 cervical cancer cells without a significant effect on the HaCat cells, showing a most significant effect on HeLa cells: reducing the initial number of cells to 40%, at starting concentration of 250 nM and 10% at 1250 nM. Data are presented as mean ± SD (*n*  =  3); not significance (n.s.) = *P*  >  0.05; *P  <  0.05, **P  <  0.01. One-way ANOVA test using the Prism program was used for statistical analysis. (**B**) HPV-negative (HaCat), HPV 18-positive (HeLa), and HPV 16-positive (CaSki) cells were treated with 500 nM of human IKKβ inhibitor, IMD 0354, or with DMSO as control. After 3 h cells were trypsinized and stained with Propidium Iodide, and the cell cycle profiles were analysed by FACS. n =  4. Representative cell cycle profiles are shown for each cell type and condition. (**C**) The cell cycle FACS profiles were analysed to determine the number of cells in subG1 (i.e. apoptotic), G1, S and G2M phases of the cell cycle. The histogram shows the results from four experiments expressed as the fold change in cell number upon IMD 0354 treatment, relative to DMSO treatment. Standard deviations are shown. The assay was repeated 4 times, n = 4.

IKKβ is a serine/threonine kinase that is highly conserved between flies and humans. It regulates the innate immune pathway by activating NF-κB, which in turn activates the expression of antimicrobial peptides to fight against pathogens^41–43^. To determine whether IKKβ also has a functional link to E6 + UBE3A in human cells, we assessed the effect of the synthetic IKKβ inhibitor IMD 0354 (N-(3,5-Bis-trifluoromethylphenyl)-5-chloro-2-hydroxybenzamide)^44^ on the growth of HaCat (HPV−ve), HeLa (HPV 18 +ve), CasKi (HPV 16 +ve), and SiHa (HPV 16 +ve) cells. IMD 0354 is a non–adenosine triphosphate–binding (ATP-binding) competitive selective IKKβ inhibitor that prevents ATP attachment to IKKβ^38^. Different concentrations of the inhibitor were tested ranging from 250, 500, 750, 1000, to 1250 nM. IMD 0354 reduced growth of all four cell types; HeLa cells were most strongly affected, with a significant effect starting at the lowest concentration of 250 nM. While IMD 0354 had a significant effect on the HPV 18 and 16 positive cells, it had a minor effect on the growth of HaCat cells that lack HPV (Fig. 2A). This result suggested that the IKKβ-mediated mechanism of E6 + UBE3A-induced cellular abnormalities is conserved between humans and fruit flies. To understand whether the reduced cell number resulting from inhibition of IKKβ is due to arrest in cell cycle or to apoptosis, we treated these cells with 500 nM of IMD 0354 and analyzed their cell cycle profile 3 h post treatment. We found no increase in apoptotic cell death for HeLa and CaSki cells in comparison with HaCat cells. However, the majority of cells were found to exhibit a G1 cell cycle arrest (Fig. 2B,C). This finding indicated that the reduction in cell numbers upon IKKβ inhibition is due to a halt in cell growth and not to an increase in apoptotic cell death.

### Reducing IKKβ suppressed the cellular defects caused by co-expression of E6 and hUBE3A

As reduction in IKKβ suppressed the rough eye phenotype caused by co-expression of E6 and hUBE3A, we examined the eye tissue of these flies 40 h after puparium formation, the time point at which the E6 + hUBE3A effect becomes apparent. In the normal pupal eye, each ommatidium consists of eight photoreceptor cells covered by four glial-like cells or cone cells and two primary pigment cells. Neighboring ommatidia are separated from each other by a lattice of secondary and tertiary pigment cells and the sensory bristle cells. This interweaving lattice of pigment cells organizes the ommatidial array into a precise pattern of repeated hexagons^45^.

**Figure 3:**
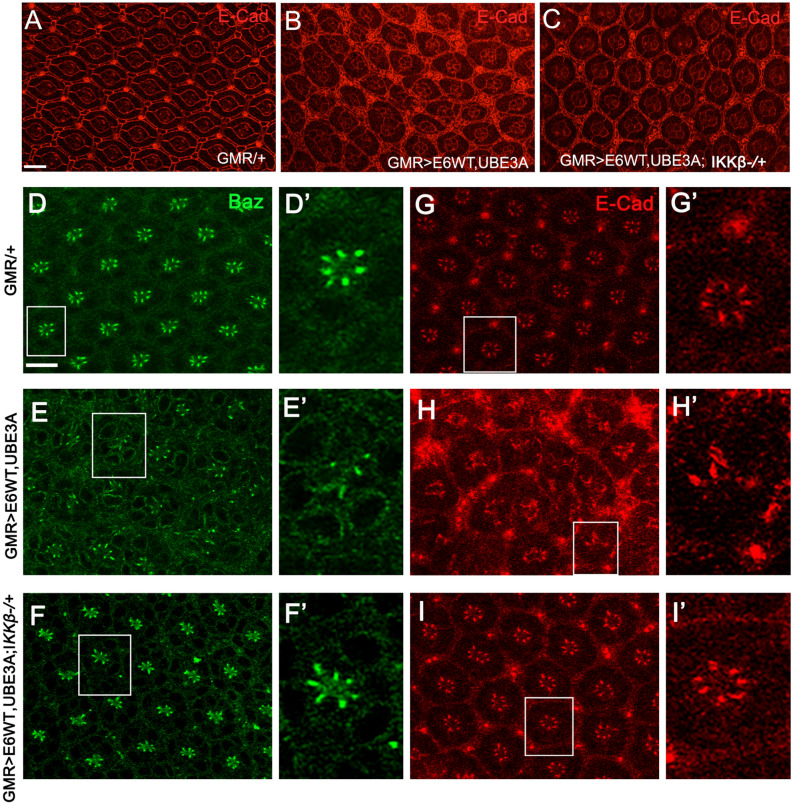
Reduction of IKKβ suppresses the cellular abnormalities and restores the cell polarity and junctional integrity disrupted by E6 + UBE3A co-expression. (**A–C**) Pupal eyes showing E-Cad immunolabeling. (**A**) Expression of GMR-Gal4, showing a normal stereotype pattern of ommatidia. (**B**) Co-expression of E6 and UBE3A causes severe abnormalities, including increase in number of pigment cells, bristle cells, and fusion of neighboring ommatidia. (**C**) A mutated copy of the IKKβ gene suppresses the E6 + UBE3A abnormalities. (**E–H′**) Co-expression of E6 and UBE3A causes disruption of cell polarity and junctional complex, as shown by loss of Bazooka (Baz) and E-Cad from photoreceptor cells. This is in comparison with (**D–G′**) where Baz and E-Cad are both localized correctly in each photoreceptor. (**F–I′**) A mutated copy of IKKβ gene in eyes expressing E6 and UBE3A restores the polarity and junctional integrity as is shown by the correct localization of Baz and E-Cad. Scale bars indicate 10 µm. Insets are digitally magnified 200%. Results shown are representative of those of 5 different flies.

Pupal eyes expressing E6 and hUBE3A exhibit severe morphological defects, including fusion of neighboring ommatidia, increase in the number of pigment cells and cone cells, and a severe alteration in the stereotyped pattern of ommatidia. In comparison, removing a single genomic copy of IKKβ (*GMR*>*E6/hUBE3A; IKKβ*^+*/−*^) led to a strong phenotypic rescue: pigment and cone cell defects were reduced and the overall organization of the ommatidial array improved (Fig. 3A–C). These observations suggest that E6 interferes with a molecular mechanism involving IKKβ and that this mechanism plays an important role in E6-induced cellular abnormalities.

### Reducing IKKβ suppressed the junctional and polarity defects caused by co-expression of E6 and hUBE3A

We have previously shown that E6, in cooperation with UBE3A, perturbs the integrity of junctional and polarity complexes^35^. Therefore, we hypothesized that reducing IKKβ activity might suppress these defects. Immunolabeling for junctional marker E-cadherin and polarity marker Bazooka (the homolog of human Par-3) revealed that, in comparison with pupal eyes expressing E6 and UBE3A, in which both the junctional and polarity complexes were perturbed in ommatidia (Fig. 3 E–H′), *GMR*>*E6/hUBE3A; IKKβ*^+*/−*^ pupal eyes showed no disruption of junctional and polarity complexes and the integrity of these complexes was restored to the extent seen in control eye tissues (Fig. 3F–I′ compared to D–G′). These observations suggest that alterations in IKKβ significantly contribute to the E6-induced cellular junctional and polarity disorganization.

### Reduced IKKβ activity suppressed E6 + hUBEA-induced degradation of PDZ domain proteins

**Figure 4:**
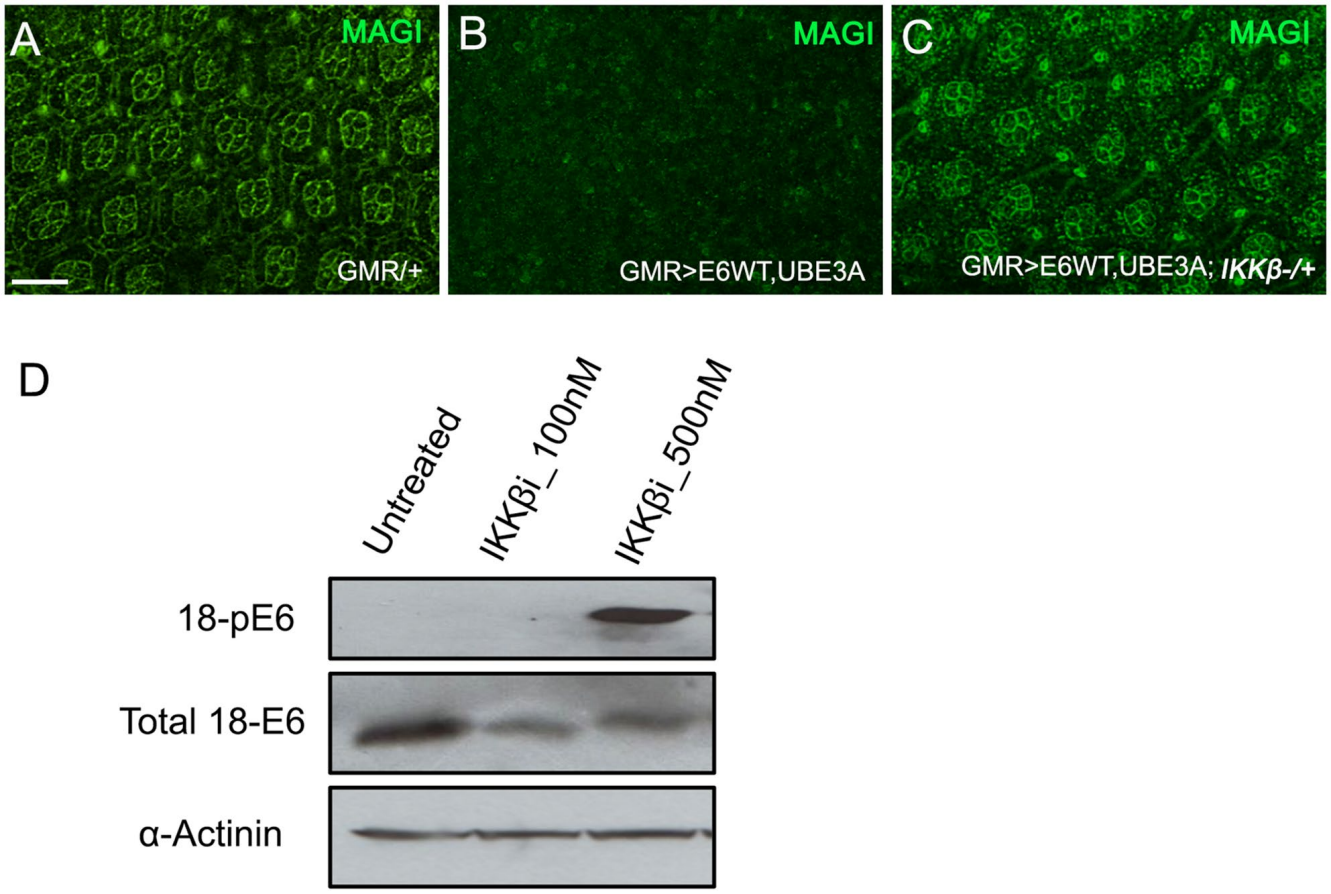
Reduced activity of IKKβ causes hyperphosphorylation of E6 and suppression of E6 + UBE3A-mediated degradation of PDZ domain protein, Magi. (**A**–**C**) Pupal eyes showing Magi immunolabeling. (**B**) Co-expression of E6 + UBE3A results in loss of Magi from cone cells and pigment cells compared with (**A**) in which only the Gal4 driver is expressed. (**C**) A mutated copy of the IKKβ gene suppresses the E6 + UBE3A-mediated loss of Magi. (**D**) Western blot of HeLa cell extracts shows that inhibition of IKKβ using a concentration of 500 nM of the inhibitor IMD 0354 results in phosphorylation of E6; compare that to lack of E6 phosphorylation in HeLa cells grown with no inhibitor (untreated) or with a low concentration of 100 nM. Scale bar indicates 10 µm. Results shown in (**A**–**C**) are representative of those of 5 different flies. Result shown in (**D**) is a representative of n = 3.

Proteasomal degradation of PDZ domain-containing proteins, including Magi, Dlg, and Scribble, was shown to be crucial for the cancerous effect of HPV 16 and 18 E6^25^. We have previously shown that HPV 18 E6, with the addition of human UBE3A, targets the fly counterparts of these proteins for ubiquitin-mediated proteasomal destruction^35^. As a reduction in IKKβ levels suppressed the cellular defects caused by E6 plus hUBE3A, we asked whether the E6-mediated degradation of PDZ domain proteins was altered. To address this question we examined the level of Magi, as Magi has been identified as a major degradation target of HPV18 E6 in human and *Drosophila*^19,35^. Immunolabeling of pupal eyes for Magi revealed that, whereas *GMR*>*E6/hUBE3A* eyes exhibited a complete loss of Magi, *GMR*>*E6/hUBE3A; IKKβ*^+*/−*^ pupal eyes exhibited no detectable loss of Magi (Fig. 4A–C). This result suggests that reducing IKKβ activity suppresses the E6-induced degradation of Magi, and that rescue of Magi degradation is likely to play a role in suppression of E6-induced cellular defects.

### Reducing IKKβ resulted in hyperphosphorylation of E6

Phosphorylation of the HPV 18 E6 PBM was previously demonstrated to block its interaction with PDZ domain proteins Dlg and Magi^37,46,47^. To assess whether phosphorylation-mediated regulation of E6 plays a role in suppressing Magi degradation, we treated cells expressing HPV 18 E6 with the IKKβ inhibitor IMD 0354 (at 100 and 500 nM) and compared with untreated cells for E6 phosphorylation. Western blot analysis was performed using an antibody to detect phosphorylated E6. We found that inhibition of IKKβ resulted in extensive phosphorylation of E6, which was absent in untreated cells (Fig. 4D). This result is consistent with previous findings, and suggests that the lack of Magi degradation in cells expressing E6 + hUBE3A with mutated IKKβ could be due to the loss of PDZ recognition by E6.

### Reducing IKKβ suppressed the cooperative effect of Ras and E6 + hUBE3A

**Figure 5:**
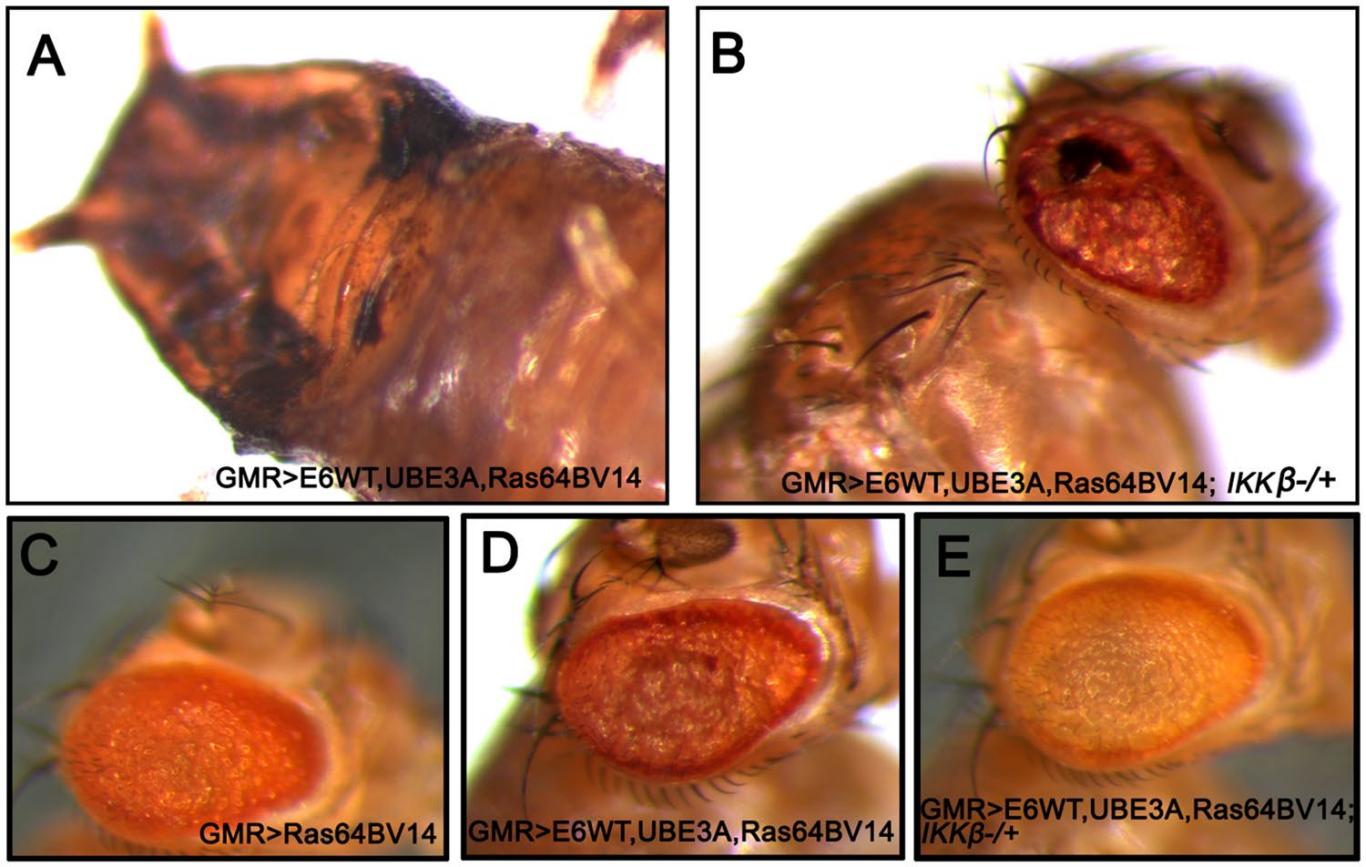
Reduction of IKKβ suppresses the cellular transformation caused by cooperative action of Ras and E6 + UBE3A. (**A**,**B**) Expression of transgenes at 25 °C. (**B**) A mutated copy of IKKβ gene in eyes expressing E6, UBE3A and an oncogenic Ras, suppresses pupal lethality and the severe defects caused by cooperative action of E6, UBE3A and oncogenic Ras (**A**). (**C**–**E**) Expression of transgenes at 22 °C. (**C**) Expression of oncogenic Ras in the eye causes cellular transformation. (**D**) Co-expression of E6 and UBE3A with oncogenic Ras enhances the Ras phenotype. (**E**) A mutated copy of IKKβ gene suppresses the severe eye abnormalities mediated by the synergistic effect of oncogenic Ras, E6 and UBE3A. Results shown are representative of those of 5 different flies.

Previous studies have shown that the HPV oncogenes E6 and E7 alone are insufficient to direct oncogenic transformation, and that other factors including genetic alterations contribute to HPV-induced tumorigenesis in humans and mice^48^. Mutations in Ras family proteins have been implicated in HPV-related cancers, and we have previously shown that cooperation between E6 and oncogenic isoforms of Drosophila Ras (Ras64B^V14^) promotes cellular transformation and malignancy in fly epithelia^35,48–50^. Therefore, we asked whether reducing IKKβ activity can suppress the cellular transformation caused by the cooperation of oncogenic Ras and E6. Co-expression of oncogenic Ras, Ras64B^V14^ with E6, and hUB3A in eye imaginal discs (*GMR*>*E6/hUBE3A/Ras64B*^*V14*^) at 25 °C resulted in overgrowth and pupal lethality (Fig. 5A). However, when the level of IKKβ was reduced (*GMR*>*E6/hUBE3A/Ras64B*^*V14*^*; IKKβ*^+*/−*^), the lethality was rescued and flies developed to adulthood. These adult flies, however, still exhibited significant abnormalities in eye morphology, suggesting incomplete rescue (Fig. 5B).

Gal4 activity is reduced at lower temperatures^51^. At 22 °C, *GMR*>*E6/hUBE3A/Ras64B*^*V14*^ flies developed to adulthood, exhibiting enhanced transformed eye morphology in comparison to expression of oncogenic Ras alone (Fig. 5D compared to 5C). Reducing IKKβ activity significantly suppressed the transformed eye morphology in these conditions (Fig. 5E), further demonstrating that IKKβ is important for the cooperative action of E6 and oncogenic Ras.

## Discussion

In this study we identify IKKβ as a mediator of the HPV 18 E6 and hUBE3A-induced cellular defects in both fly and human cancer models. This is consistent with previous studies demonstrating that HPV 16 E6 interacts with components of the innate immune pathway, including IKKβ, and it activates the NF-κB transcription factor^52,53^. The role of the innate immune system in HPV infection and cancer progression is not well understood. Activation of the innate immune system, particularly the Toll pathway, has been associated with regression and clearance of HPV 16 infection^54^. This is consistent with other studies demonstrating that HPV 16 oncogenes repress the expression of a key innate immune sensor, Toll like receptor 9 (TLR9)^55^, suggesting that persistent infection may reflect a defect in the host innate immune system. Conversely, activation of the innate immune system, and consequent inflammation, contributes to HPV-induced tumor progression^56^. Several studies have shown an increase in the level of inflammatory cytokines in individuals with reduced HPV clearance^57–59^. Additionally, upregulation of several TLRs and components of the NF-κB signaling pathway have been implicated in HPV-related cancers. It has been suggested that the malignant phenotype of cervical cancer cells requires the function and activation of IKKβ/NF-κB signaling^60,61^. IKKβ phosphorylates the inhibitor of NF-κB, resulting in its ubiquitination and proteasomal degradation. This action of IKKβ frees the NF-κB, which in turn enters the nucleus and activates the transcription of pro-inflammatory, pro-cell proliferation and anti-apoptotic genes^62,63^. Increased expression of IKKβ and its association with an aggressive phenotype has been reported in several types of cancers including head-and-neck, ovarian and liver cancers^64–66^. It is notable that IKKβ's role in cancer is not only limited to its function in regulation of the NF-κB pathway; IKKβ can also phosphorylate p53, resulting in its ubiquitination and subsequent degradation. Inactivation or loss of p53 has been identified in more than 50% of cancers, including HPV-induced cancers: high-risk HPV E6s target p53 for ubiquitination and proteasomal degradation^14,67^. IKKβ-mediated loss of p53 can be suppressed by inhibition of IKKβ in cancer cells^68,69^. The inhibitory function of IKKβ on p53 was further found in HPV38E6E7 human keratinocytes. IKKβ phosphorylates and stabilizes a dominant-negative inhibitor of p53, ∆Np73$\alpha ,$ resulting in repression of p53-regulated genes such as p21. This inhibitory effect of IKKβ on p53 can be suppressed upon its inactivation, which results in destabilization and degradation of ∆Np73$\alpha$^70^. Another phosphorylation target of IKKβ is fork-head transcription factor FOXO3a. IKKβ has been found to stimulate cell cycle progression and proliferation of breast cancer cells, through phosphorylation of FOXO3a, leading to its exclusion from nucleus and subsequent degradation in the proteasome. This function of IKKβ could be further overridden by the overexpression of FOXO3a^71^. All these findings suggest that IKKβ may contribute to HPV-induced cellular abnormalities in several ways, some through the innate immune pathway, and some independent of it.

IKKβ inhibitors have served as potent anti-tumor agents inhibiting cell proliferation and invasiveness, as well as inducing cell death. Their activity has been demonstrated in several cancer types including breast, colon, ovarian, oral, prostate, liver, melanoma, and leukemia^65,66,72–76^. One such inhibitor is IMD 0354, which selectively inhibits IKKβ by preventing ATP attachment to IKKβ. This will in turn block phosphorylation of IκBα, thus preventing nuclear translocation and activation of NF-κB^38^. Although IMD 0354 was initially designed to inhibit inflammation^77^, it has recently proved to have strong anticancer properties^78,79^ in several cancers. IMD 0354 inhibits breast cancer cell proliferation, progression of mast cells, and it induces apoptosis in prostate, lung and colon cancers^44,80–82^. In our study we found that IMD 0354 inhibited the growth of HPV 16+ and 18+ cervical cancer cell lines. This result is consistent with previously reported function of IMD 0354 in cancer cells and hence suggests a therapeutic potential for IKKβ inhibitors to treat HPV-induced cancers. Although both HPV16+ and HPV18+ cervical cancer cell lines responded to IMD 0354, however, IMD 0354-mediated inhibition of IKKβ exhibited greater effectiveness on HeLa cells (HPV 18+) compared with SiHa and CaSki cells (HPV 16+). It has previously been demonstrated that these cell lines differ in a number of molecular pathways, and in their response to treatments such as therapeutic agents that induce cell death^83–85^. HeLa cells exhibit greater apoptotic cell death in response to cisplatin than SiHa and CaSki cells do, a difference that is due to the higher levels of p53 and p21 in HeLa cells^84^. Additionally, these cell lines show distinct proteomic profiles for pathways connected to p53 activation, mitochondrial function and oxidative stress^85,86^. These findings suggest that the stronger effect of IMD 0354 in HeLa relative to SiHa and CaSki cells could in part be due to their difference in molecular pathways affected by IKKβ. Additionally, IMD 0354 caused an extensive phosphorylation of E6, which was absent in untreated cells. This finding is intriguing, as phosphorylation of E6 on the PDZ binding motif results in disruption of its interaction with PDZ domain-containing proteins^46,47^. This is consistent with our results, as degradation of Magi was inhibited when IKKβ was reduced. It would be of interest to understand the mechanism by which inhibition of IKKβ results in E6 hyperphosphorylation.

In summary this study suggests an important role for IKKβ in E6-induced cellular abnormalities and that targeting IKKβ could be a potential therapeutic option for cervical cancer and, perhaps, for other HPV-related cancers for which there is currently no effective treatment.

## Supplementary Information

Supplementary Information.
